# In Vitro Antioxidant, Antimicrobial, Anticoccidial, and Anti-Inflammatory Study of Essential Oils of Oregano, Thyme, and Sage from Epirus, Greece

**DOI:** 10.3390/life12111783

**Published:** 2022-11-04

**Authors:** Erasmia Sidiropoulou, Virginia Marugán-Hernández, Ioannis Skoufos, Ilias Giannenas, Eleftherios Bonos, Kensilandia Aguiar-Martins, Diamanto Lazari, Theodora Papagrigoriou, Konstantina Fotou, Katerina Grigoriadou, Damer P. Blake, Athina Tzora

**Affiliations:** 1 Laboratory of Nutrition, School of Veterinary Medicine, Faculty of Health Sciences, Aristotle University of Thessaloniki, 54124 Thessaloniki, Greece; 2 Department of Pathobiology and Population Sciences, Royal Veterinary College, University of London, Hertfordshire AL9 7TA, UK; 3 Laboratory of Animal Production, Nutrition and Biotechnology, Department of Agriculture, School of Agriculture, University of Ioannina, Kostakioi Artas, 47100 Arta, Greece; 4 Laboratory of Pharmacognosy, School of Pharmacy, Faculty of Health Sciences, Aristotle University of Thessaloniki, 54124 Thessaloniki, Greece; 5 Laboratory of Animal Health, Food Hygiene and Quality, Department of Agriculture, University of Ioannina, 47132 Arta, Greece; 6 Institute of Plant Breeding and Genetic Resources, Hellenic Agricultural Organization—DEMETER, Thermi, 57001 Thessaloniki, Greece

**Keywords:** oregano, thyme, sage, antimicrobial, anticoccidial, anti-inflammatory

## Abstract

**Simple Summary:**

In recent years, consumers’ concern over the use of synthetic antioxidants and antibiotics in food is on the rise, prompting extensive research for alternatives of natural origin. Three essential oils from aromatic plants used in Greek traditional medicine were tested for their antioxidant, anti-inflammatory, and antimicrobial activity in order to determine their applicability as feed additives. The in vitro results showed that plants originating from the western part of Greece, the area of Epirus, possess potent anticoccidial, antimicrobial, anti-inflammatory, and antioxidant activity.

**Abstract:**

*Origanum vulgare subsp. hirtum, Thymus vulgaris*, and *Salvia fructicosa* are aromatic plants commonly found in Mediterranean countries and are traditionally used in Greece as a remedy for humans, since they are well known as potent antibacterial, antioxidant, and anti-inflammatory agents. Essential oils (EOs) derived from plants cultivated in the mountainous region of Epirus, Greece, were investigated for their inhibitory activity against key microorganisms with relevance to avian health, while also assessing their antioxidant and anti-inflammatory activity. The total phenolic content (TPC) of the EOs was estimated according to the Folin–Ciocalteu method, while the antioxidant capacity was tested through the EOs’ ability to scavenge free radicals by means of the DPPH, ABTS, and FRAP assays. Antibacterial and anti-inflammatory effects were examined by the agar disc diffusion method and the lipoxygenase (LOX) inhibition test, respectively. Furthermore, the EOs’ ability to inhibit the invasion of sporozoites of *Eimeria tenella* (Wisconsin strain) along with any toxic effects were assayed in Madin–Darby bovine kidney (MDBK) cells. The antioxidant activity of the EOs was observed in descending order: oregano > thyme > sage. The antimicrobial effects of thyme and oregano were equivalent and higher than that of sage, while the anti-inflammatory effect of thyme was higher compared to both sage and oregano. The intracellular invasion of sporozoites was evaluated by the detection of *E. tenella* DNA by qPCR from cell monolayers harvested at 2 and 24 h post-infection. Parasite invasion was inhibited by the addition of oregano essential oil at the concentration of 100 μg/mL by 83% or 93% after 2 or 24 h, respectively, and was higher compared to the addition of thyme and sage, which had similar effects, but at a less intensive level. The cytotoxic assessment of all three essential oils revealed that they had no effect on MDBK cells compared to dimethyl sulfoxide (DMSO), used as the control substance. The supplementation of oregano, thyme, and sage essential oils had a potent antioxidant, anti-inflammatory, antimicrobial, and anticoccidial in vitro effect that is comparable to synthetic substances or approved drugs, justifying the need for further evaluation by in vivo studies in broilers reared in the absence of antimicrobial and anticoccidial drugs or synthetic antioxidant and/or anti-inflammatory compounds.

## 1. Introduction

Calls to screen natural compounds to discover solutions for the control of avian pathogens and to preserve oxidative stability in meat products have increased in response to the overuse of antibiotics [1]. Plant-derived secondary metabolites of importance include phenolic acids, flavonoids, terpenes, and volatile compounds. Essential oils, belonging to the latter group, along with some of their constituents such as carvacrol, eugenol, or thymol have become well known for their antioxidant and/or antibacterial activity and several phenolic compounds have been investigated for their ability to suppress microbial growth [2,3,4].

Oregano, thyme, and sage have frequently been exploited for preservative, culinary, and medicinal functions throughout history [2,5,6]. Greek oregano (*Oregano vulgare* subsp. *hirtum*; family Lamiaceae) offers multiple medicinal qualities including anti-inflammatory, antioxidant, analgesic, hepato-, gastro-, and neuroprotective properties, while its essential oil (ΕO) contains a high concentration of active ingredients, acquiring a high ranking as one of the best quality herbs in the world with significant commercial value [7,8]. The antimicrobial activity of oregano essential oil (OEO) has been thoroughly investigated and attributed to its high content in carvacrol (up to 90%), thymol, γ-terpinene, and p-cymene, influenced by the area of cultivation, harvesting season, and species [9,10]. Moreover, it has been observed to possess excellent antimicrobial and antioxidant capacity [1,11]. Thus, it is potent not only against Gram-negative bacteria such as *Escherichia coli*, *Salmonella* spp., or *Proteus*, but also Gram-positive bacteria, including lactic acid bacteria such as *Lactobacillus*, *Leuconostoc*, *Pediococcus*, *Lactococcus*, and *Streptococcus.* The antibacterial action of EOs stems from their ability to inhibit bacterial growth by altering cell membrane permeability and reducing bacterial toxin production, as they are viscous lipophilic liquids [12,13,14,15]. Additionally, OEO may limit the oxidation processes of raw meat and other food preparations because of its antioxidant properties [16,17]. Both antioxidant and antimicrobial effects may delay food from becoming off-flavor, rotten, or deteriorating due to the production of reactive oxygen species (ROS) and the proliferation of harmful microorganisms [18,19,20,21]. Likewise, many studies support the notion that OEO can reduce lipid peroxidation in meat, liver, and serum when added to broiler chicken feed [22,23]. In a recent study, OEO was shown to be useful to treat inflammation and promote wound healing [24]. Although there is a lot of information regarding the antibacterial activity of OEO, limited evidence exists on its efficacy and/or effectiveness against parasites, including a small number of in vitro studies that revealed the potential of OEO against parasite activity [25]. OEO was shown to inhibit *Cryptosporidium parvum* infectivity in HCT-8 cells without modulating sporozoites invasion [25] and exerted an antiparasitic capacity against protozoa such as *Plasmodium falciparum*, trypomastigote forms of *Trypanosoma* spp. and amastigotes of *Leishmania donovani* [26]. Interestingly, both carvacrol, the main phenolic monoterpenoid in OEO, and thymol in thyme essential oil exhibited the same antiprotozoal potency. They have also been tested against coccidia in vivo and in vitro [27,28].

*Thymus vulgaris* is recognized as a high-yielding source of essential oil, providing a minimum of 12 mL/kg, and is widely used for pharmaceutical and culinary purposes. Thyme essential oil (TEO) is a rich source of a wide range of aromatic bioactive components such as thymol and carvacrol, with a noted role as an antioxidative and antimicrobial agent [29]. TEO components include thymol, γ-terpinene, p-cymene, linalool, geraniol, and carvacrol, all of which possess antimicrobial properties as shown in various studies [29,30,31]. TEO has been screened against several common food-related bacteria including *Staphylococcus aureus*, *Pseudomonas aeruginosa*, *Salmonella typhimurium*, *Escherichia coli*, *Klebsiella pneumoniae*, and *Enterococcus faecalis*, and has been observed to exhibit strong antimicrobial properties under in vitro conditions [32]. TEO, alone or in combination with other EOs, has also been reported to act against Gram-negative and/or positive bacteria in food-related preparations [33]. For these purposes, EOs can be directly added to the food surface and some edible films used in food preparations may serve as carriers [34,35]. Regarding TEO’s antiparasitic action, previous studies have revealed contradictory results, with the proliferation of *Trypanosoma brucei* being compromised when TEO was added in HL-60 cells, but not exhibiting any antiparasitic potency against *Leishmania* spp. [36].

Another essential oil of interest is derived from *Salvia fructicosa* (family Lamiaceae), commonly known as sage, which is abundant in southern Europe. Sage has been widely used as a food herb and medicine since ancient times. Numerous studies corroborate its beneficial biological qualities, including antioxidant, anti-inflammatory, and antimicrobial properties [37,38]. Different chemical components have been shown to be responsible for these activities [39,40]. Due to its high content of phenolics (such as polyphenols and flavonoids) and terpenoids, sage EO (SEO) exhibits antioxidant activity [40,41]. The most important components in SEO include linalool and terpinene. In vitro studies have proven that sage extracts positively affect and protect cultured cells from inductive oxidative stress. SEO was reported to reduce DNA damage related to inflammation and its components inhibited both lipoxygenase and acetylcholinesterase enzymes that are related to inflammatory and other chronic illnesses [41]. Moreover, sage was observed to protect HepG cells [42].

The purpose of the present study was to focus on the evaluation of the in vitro antioxidant, antimicrobial, anti-inflammatory, and antiparasitic activity of the essential oils obtained from Greek oregano, thyme, and sage plants.

## 2. Materials and Methods

### 2.1. Plant Sources and Essential Oil Extraction

**Figure 1:**
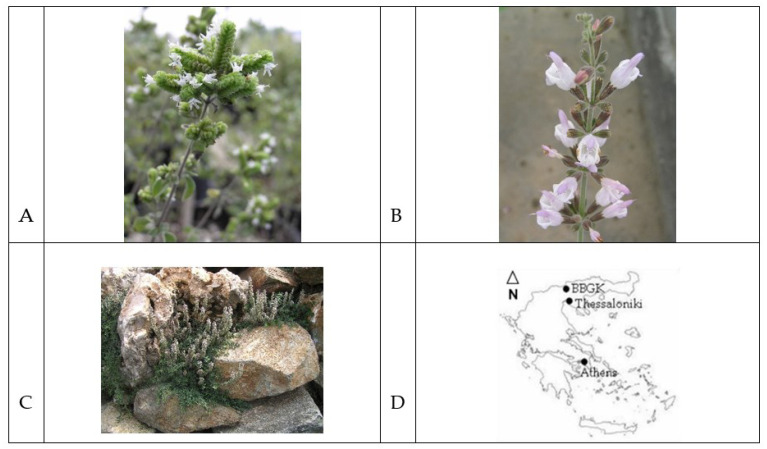
Photos of oregano, (**A**), Salvia, (**B**), Thyme, (**C**), and the map area BBGK in Greece, (**D**).

Plant material of native Greek *Origanum vulgare* subsp. *hirtum* L. (oregano, IPEN (International Plant Exchange Network) accession number GR-1-80 BBGK- 03,2107), *Salvia fructicosa* (sage, IPEN accession number GR-1-80 BBGK- 04,2411) and from the cultivar *Thymus vulgaris* L. var Varico 3 (thyme) are ex situ maintained at the collection of the Balkan Botanic Garden of Kroussia 84 (41°05′44.3′′ N 23°06′33.7′′ E) of the Institute of Plant Breeding and Genetic Resources, Hellenic Agricultural Organization—DEMETER, in Greece (Figure 1). From these mother plants, young plants were produced asexually by cuttings and were provided to the company “Aromata Epirus”, (Palaiohori, Filiates Thesprotia, Epirus, Greece) where they were cultivated in the fields in autumn. After 1.5 years, when the plants were in full blossom, the leaves and flowers were collected and dried and the dried material was transferred to the Laboratory of Pharmacognosy, School of Pharmacy, Aristotle University of Thessaloniki, where it was submitted to hydrodistillation for 2 h using a modified Clevenger-type apparatus with a water-cooled oil receiver to reduce hydrodistillation overheating artifacts. The volatiles were trapped in 5 mL gas chromatography-grade n-hexane, according to the standard procedure described in European Pharmacopeia, dried over anhydrous sodium sulfate, and kept in closed, air-tight Pyrex containers at −4 °C until use in the in vitro trials. The volatile constituents of the essential oils were analyzed by gas chromatography–mass spectrometry (GC-MS) analysis, using a Shimadzu GC-2010-GCMS-QP2010, as previously described [30]. Authentic compounds (Fluka, Sigma-Aldrich, Taufkirchen, Germany) were used for co-chromatography comparison.

### 2.2. Determination of EOs’ Active Compounds

The composition of the volatile constituents obtained by hydrodistillation was established by gas chromatography–mass spectrometry (GC–MS) analysis. The analyses of the essential oils were performed on a Shimadzu GC-2010-GC/MS-QP2010 system operating at 70 eV. This was equipped with a split/splitless injector (230 °C) and an HP INNOWAX capillary column (30 m × 0.25 mm i.d., film thickness 0.25 μm). The temperature program ranged from 50 °C (20 min) to 260 °C, at a rate of 3 °C/min. Helium was used as a carrier gas at a flow rate of 1.0 mL/min. The injection volume of each sample was 1.0 μL. The relative percentage amounts were calculated from total ion chromatograms (TIC) by the computer. Arithmetic indices for all compounds were determined according to van den Dool and Katz [43], using n-alkanes as standards. The identification of the components was based on the comparison of their mass spectra with those of NIST21 and NIST107 and their retention indices with literature data [44,45]. Essential oils were also subjected to co-chromatography with authentic compounds (Fluka, Sigma) and their essential oil yield was expressed in mL 100 g^−1^ d.w.

### 2.3. Determination of Total Phenolic Content of EOs

The total phenolic content (TPC) of the EOs was determined using the Folin–Ciocalteu method [46], with slight modifications: 5 mg of each EO was diluted in 1 mL of absolute ethanol (EtOH), and 20 μL of each ethanolic solution was added to a test tube containing 2500 μL deionized water and 400 μL Folin–Ciocalteu phenol reagent (F9252, Sigma-Aldrich, Taufkirchen, Germany). The mixtures were kept in the dark at room temperature for 8 min. Then, 500 μL of Na_2_CO_3_ 7% solution was added to the tubes and the mixture was incubated in the dark at room temperature for 45 min. Each sample absorbance was measured at λ = 750 nm, using a UV-Vis spectrophotometer (UV-1700 PharmaSpec, Shimadzu, Kyoto, Japan). The total phenolic content was calculated by means of a standard curve, using standard solutions of gradually increasing concentrations (0–1.5 mg mL ^−1^, R^2^ = 0.947) of gallic acid. The results were expressed as mg of gallic acid equivalents per L of EO (mg GAE L^−1^), from the mean of three measurements ± standard error for each sample.

### 2.4. Antioxidant Assays

Several in vitro assays were employed for the assessment of the antioxidant activity of the examined EOs, namely: (i) interaction with the free stable radical DPPH (1,1-diphenyl-2-picrylhydrazyl), (ii) ABTS (2,2′-azino-bis (3-ethylbenzothiazoline-6-sulfonic acid)) radical cation decolorization assay, and (iii) FRAP (ferric-reducing antioxidant power). In all of the aforementioned assays, the EO samples were diluted in absolute EtOH at a concentration of 5 mg mL^−1^ (for the DPPH assay) or 1 mg mL^−1^ (for the FRAP and the ABTS assays). In all assays, a UV-1700 PharmaSpec spectrophotometer (Shimadzu, Kyoto, Japan) was used for the absorbance measurement. All experiments were carried out in triplicate and the results were expressed as mean ± standard error.

#### 2.4.1. Interaction with DPPH

The DPPH assay was performed by incubating 20 μL of ethanol-diluted EOs (5 mg mL^−1^) with DPPH (D211400, Sigma-Aldrich, Taufkirchen, Germany) methanolic solution (0.1 mM) in the dark, at room temperature. Each sample absorbance was measured at λ = 517 nm at two time points, t_1_ = 20 min and t_2_ = 60 min, and the percentage of radical-scavenging activity (%RSA) was calculated as follows:

%RSA = [(Ab − As)/Ab] × 100(1)

where Ab is the absorbance of the blank sample (containing EtOH instead of the EO samples) and As is the absorbance of each sample [38]. Trolox was used as a reference compound.

#### 2.4.2. ABTS Radical Cation Decolorization Assay

For the preparation of the ABTS^+^ solution, 38.4 mg of ABTS (A1888, Sigma-Aldrich, Taufkirchen, Germany) was dissolved in 10 mL of water, along with 6.6 mg of K_2_S_2_O_8_ (potassium persulfate) (7 mM and 2.45 mM, respectively) and stored in the dark for 16 h at room temperature. The next day, the radical solution was diluted with ethanol to an absorbance of 0.70 ± 0.02 at *λ* = 234 nm. The protocol of Zhen et al. [47] was followed, with slight modifications: 30 μL of EO sample (diluted in EtOH) was added to 2970 μL of ABTS^+˙^ solution and the mixture was left to stand in the dark for 6 min at room temperature. The percentage of discoloration (and, hence, the reducing activity of the samples) was calculated in the same manner as in the DPPH assay (see Equation (1)). The blank sample consisted of 100% EtOH, and Trolox was used as a reference compound. The results were expressed in the same way as the DPPH assay, i.e., %RSA ± standard error.

#### 2.4.3. Ferric-Reducing Antioxidant Power (FRAP) Assay

The ferric-reducing antioxidant power (FRAP) of the samples was determined according to the protocol of Benzie and Devaki [48]: 2900 μL of the FRAP solution, heated at 37 °C, was added to a test tube containing 100 μL of EO sample (or pure EtOH for the blank sample) and the mixture was vortexed and left in the dark for 30 min at room temperature. At the end of the incubation time, the samples’ absorption was measured at 593 nm, and their FRAP values were expressed as μmol ascorbic acid L^−1^ (μmol AsA L^−1^) based on a calibration curve (0–2,000 μmol AsA L^−1^, R^2^ = 0.996). The FRAP solution consisted of acetate buffer (300 mM, pH 3.6), FeCl_3_ (20 mM in ultrapure water), and TPTZ (2,4,6-tripyridyl-S-triazine, A17201.06, Alfa Aesar, Sigma-Aldrich, Taufkirchen, Germany) (10 mM in 40 mM HCl) at a ratio of 10:1:1, freshly prepared before each assay.

### 2.5. Anti-Inflammatory Assay: Soybean Lipoxygenase Inhibition

Each sample’s ability to inhibit soybean lipoxygenase in vitro was used as a measurement of their anti-inflammatory potential by means of the FOX test, an assay based on the formation of a deep red-brown complex between xylenol orange and Fe^3+^ in acidic conditions, which are, in turn, formed by the hydroperoxides resulting from the oxidation of linoleic acid by soybean lipoxygenase. For this purpose, the protocol of Ondua et al. [49] was followed, with reagent volumes adjusted for a spectrophotometer: the EO samples were first diluted in dimethyl sulfoxide (DMSO) and subsequently in Tris–HCl buffer (50 mM, pH 7.4) at a final concentration of 0.5 mg mL^−1^. Quercetin was used as a positive control (final concentration 1 mg/mL). Then, 200 μL of the EOs (or quercetin) was added to test tubes and mixed with Tris–HCl buffer (200 μL) and 400 μL of LOX enzyme (L7395-15MU, Sigma-Aldrich, Taufkirchen, Germany) dissolved in ice-cold Tris–HCl buffer (final concentration 0.2 U mL^−1^). The mixture was incubated at room temperature for 5 min, and afterwards, 400 μL of linoleic acid (i.e., the enzyme’s substrate) dissolved in Tris–HCl (final concentration 140 μM) was added to the assay mixture, which was then left to stand in the dark for 20 min at room temperature. Finally, 1,000 μL of freshly prepared FOX reagent (xylenol orange (100 μM), FeSO_4_ (100 μM), and H_2_SO_4_ (30 mM) in MeOH_(aq)_ 90%) were added to the tubes and the mixture was incubated for 30 min. The negative control contained a mixture of DMSO/Tris–HCl buffer instead of EOs, whereas in the blank samples, linoleic acid was added to the assigned tubes at the end of the third incubation period, just before the absorbance measurement, at 560 nm. Each EO sample had its own negative control and blank. Each sample’s inhibitory activity was calculated according to the following formula:

%LOX inhibition = [(Ac − As)/Ac] × 100(2)

(Ac = absorbance of the negative control and As = absorbance of EO sample absorbance of the corresponding blank). The results are expressed as the mean of the three measurements ± standard error for every EO.

### 2.6. Antimicrobial Capacity

#### 2.6.1. Antibacterial Activity with Disk Diffusion Method

All of the reference bacterial strains that were used for the experiment were purchased from American Type Culture Collection (ATCC). The antibacterial activity was examined by the agar disc diffusion method (Clinical and Laboratory Standards Institute CLSI 2012, doc M02-A11). The three essential oils were provided to the Laboratory of Animal Production, Nutrition, and Biotechnology, Department of Agriculture, School of Agriculture, University of Ioannina, to assess the antibacterial properties of the EOs test samples. Four ATCC bacterial strains were used: *S. aureus* ATCC25923, *E. coli* ATCC 25922, *E. coli* ATCC 35,218 and *Lactobacillus fermentum* ATCC 9338, obtained from the microbial collection of the Laboratory of Food Hygiene. Pure EOs were diluted to 50%, 20%, and 5% concentration in DMSO 5% (*v*/*v*). Forty-eight hours before the study, the bacterial strains were revitalized and checked for purity. Bacterial suspensions of each bacterial ATCC strain in saline solution of 0.5 McFarland (density of approximately 1.5 × 10^8^ CFU mL^−1^) turbidity were streaked onto Mueller–Hinton agar (MHA) with a sterile swab. A sterile filter disk (diameter 6 mm, Whatman paper N. 1) was impregnated with EOs (15 µL/disk). The inoculated MHAs were incubated aerobically at 37 °C for 18–24 h for *S. aureus* ATCC25923, *E. coli* ATCC 25,922, and *E. coli* ATCC 35,218 and anaerobically at 37 °C for 48 h for *Lactobacillus fermentum* ATCC 9338. The antimicrobial activity was evaluated by measuring the zones of growth inhibition. Penicillin G (10 µg, Oxoid, Basingstoke, UK) was used as the positive control for Gram-positive bacteria, while enrofloxacin (5 µg, Oxoid, Basingstoke, UK) was used as the positive control for Gram-negative bacteria. The experiment was performed in triplicate.

#### 2.6.2. Determination of MIC

The minimum inhibitory concentration (MIC) was determined using the broth microdilution method according to the Clinical and Laboratory Standards Institute doc M07Ed11, with modifications. Here, 200 µL of each pure EO was pipetted in the first well of rows A–F of a 96-well plate, and then 100 µL of double-strength Müller–Hinton broth (MHB) containing 5% DMSO was dispensed into wells (2 to 12) of each column. Following this, 100 µL of each EO from the first column was sequentially mixed in MHB in the neighboring column, achieving two-fold serial dilutions. The remaining 100 µL from the last dilution mix was discarded. Forty-eight hours before the study, the bacterial strains were revitalized on Columbia blood agar (Oxoid Limited, Basingstoke, UK) and checked for purity. A standard inoculum of 0.5 McFarland units (density of approximately 1.5 × 10^8^ CFU mL^−1^) from each tested organism was prepared in sterile saline. The bacterial suspension was diluted 1/10 in saline solution and finally 100 µL was added to each well of each column over the EOs, which were mixed in the previous stage in MHB containing 5% DMSO. Thus, the EO concentration (*v*/*v*) in the final volume in wells 1–12 was 50%, 25%, 12.5%, 6.25%, 3.125%, 1.562%, 0.781%, 0.39%, 0.195%, 0.097%, 0.048%, and 0.024%, with each well containing approximatively 1.5 × 10^6^ CFU. Positive and negative control wells were prepared for each plate, in the last row (100 µL Müller–Hinton broth and 100 µL of standardized bacterial inoculum for positive control in well H11, and 200 µL Müller–Hinton broth without essential oils for negative control in well H12). The plates were placed for incubation accordingly. The MIC was interpreted in the last well of each row where no visible bacterial growth was noticed (bacterial growth inhibition) and interpreted as *v/v* percentage of stock solution.

#### 2.6.3. Determination of MBC

The minimum bactericidal concentrations (MBC) were determined from the last three wells of each row that showed no bacterial growth after plate incubation. For this, 15 µL from the corresponding wells was spot-inoculated on blood agar plates, which were labeled with the corresponding well’s coordinates. The plates were incubated overnight at 35 °C, and colony development was followed in each spot-inoculation place. The MBC was noted for the position where no bacterial colonies developed and interpreted as *v/v* percentage of stock solution.

### 2.7. Antiparasitic Capacity of EOs

#### 2.7.1. Essential Oils

Stocks of oregano, thyme, and sage essential oils were supplied to the Department of Pathobiology and Population Sciences, Royal Veterinary College, University of London and prepared to a final concentration of 1 mg mL^−1^ in DMSO.

#### 2.7.2. Cell Culture

Madin–Darby bovine kidney (MDBK) (Sigma-Aldrich, Taufkirchen, Germany) was maintained at 37 °C–5% CO_2_ in Advanced DMEM (Gibco, Leicestershire, UK) supplemented with 2% fetal bovine serum (FBS; Sigma, Suffolk, UK) and 100 U penicillin/streptomycin (Fisher, Leicestershire, UK). Monolayers of MDBK cells were prepared in 24-well plates at 0.3 × 10^6^ cells/well and seeded ~3 h prior to infections.

#### 2.7.3. Parasites

Sporozoites of the *Eimeria tenella* Wisconsin strain were used to perform the infections. Oocyst excystation and sporozoite purification were performed as described previously [50].

#### 2.7.4. Cytotoxicity Test

Each EO was also tested for cytotoxic effects in MDBK cells using 100 μL mL^−1^ per well. Morphological changes on the cell line were observed up to 24 h after exposure and compared to the control groups (DMEM/DMSO).

#### 2.7.5. Pretreatment and Infection

Sporozoites of *E. tenella* (0.5 × 10^6^/well) were pretreated for 1 h at 41 °C–5% CO_2_ with essential oils of thyme and sage at different concentrations (100, 50, 20, and 5μg mL^−1^). DMSO (10 μL mL^−1^) and robenidine (5 μg mL^−1^) were used as untreated and inhibited controls for *E. tenella* invasion, respectively. After pretreatment, sporozoites were added to monolayers of MDBK cells (41 °C–5% CO_2_, two wells/time point/group). At 2 h and 24 h hours post infection (hpi), the infected monolayers were washed in phosphate-buffered saline (0.5 mL/well) and the cells were dissociated using 0.35 mL of RTL buffer provided with a DNeasy Blood & Tissue Kit (Qiagen, Manchester, UK) and stored at −20 °C. Two biological replicates were performed.

(3) $$\mathrm{Level}\;\mathrm{of}\;\mathrm{inhibition}\;(\%)=100\times \left(1-\frac{\mathrm{average}\;\mathrm{number}\;\mathrm{of}\;E.\;\mathrm{tenella}\;\mathrm{gDNA}\;\mathrm{in}\;\mathrm{treated}\;\mathrm{sample}}{\mathrm{average}\;\mathrm{number}\;\mathrm{of}\;E.\;\mathrm{tenella}\;\mathrm{gDNA}\;\mathrm{in}\;\mathrm{sample}\;\mathrm{treated}\;\mathrm{with}\;\mathrm{DMSO}}\right)$$

The proportion of sporozoite invasion was calculated normalizing samples with the DMSO group to evaluate the inhibition level following the method adapted to Thabet et al. [51].

#### 2.7.6. Isolation of Nucleic Acids and Real Time Quantitative PCR

Genomic DNA was isolated using a DNeasy Blood & Tissue Kit (Qiagen, Manchester, United Kingdom) according to the manufacturer’s instructions. The DNA was eluted in a final volume of 165 μL per sample. Real-time quantitative PCR (qPCR) was performed in a CFX96 Touch^®^ Real-Time PCR Detection System (Bio-Rad, Hertfordshire, UK) according to Marugán–Hernández et al. [52]. The quantification of *E. tenella* per sample used gDNA and primers targeting the *Eimeria* genus 5S rDNA. Each qPCR plate used a mix of 19 μL/well (10 μL of Evan Green, 0.5 μl of 10 μM 5S Primer and 8.5 μL molecular grade water) and 1 μL of DNA. A standard curve with serial dilutions of sporozoite DNA was also tested per plate using concentrations from 1 × 10^7^ to 1 × 10^1^ g DNA *E. tenella* genomes. All groups and the standard curve were evaluated testing three technical replicates per sample.

### 2.8. Statistical Analysis

The experimental data for antioxidant, anti-inflammatory, and antimicrobial activity were subjected to analysis of variance (ANOVA) using the statistical package SPSS version 20.0 for Windows (SPSS, Inc., Chicago, IL, USA). As the bacterial numbers were not normally distributed, they were log10 transformed to create a normal distribution prior to analysis. Tukey’s post hoc test (*p* < 0.05) was performed to assess any significant differences between the experimental treatments. All data obtained from the antiparasitic studies were analyzed using the Bio-Rad CFX Manager software (Bio-Rad). The quantification of the number of parasites was performed considering the standard deviation (SD) of Cq values for replicates, excluding SD > 0.05. The average starting quantity (SQ) values per sample were used to plot graphics. Statistical analysis was done by GraphPad (GraphPad Prism 8, CA, San Diego, USA). The Shapiro–Wilk test was used to access data normality. Differences and comparisons among groups were performed by one-way ANOVA or Kruskal–Wallis test, followed by Dunn’s multiple comparisons test. Two levels of comparison (time and dosage) were tested using normalized data and the mixed-effects model REML.

## 3. Results

### 3.1. Yield of Crude Extracts and Fractions

The hydrodistillation of oregano fresh material yielded 5.49% essential oil. The hydro-distillation of thyme fresh material yielded 4.15% essential oil, whereas the sage fresh material yielded 4.66% essential oil.

### 3.2. Chemical Composition of Tested EOs

Supplementary Figures S1–S3 provide the chromatograms for oregano, salvia and thyme essential oil, respectively. Table 1, Table 2 and Table 3 provide the detailed composition of OEO, TEO, and SEO, respectively.

### 3.3. Total Phenolic Content (TPC) of EOs

**Figure 2:**
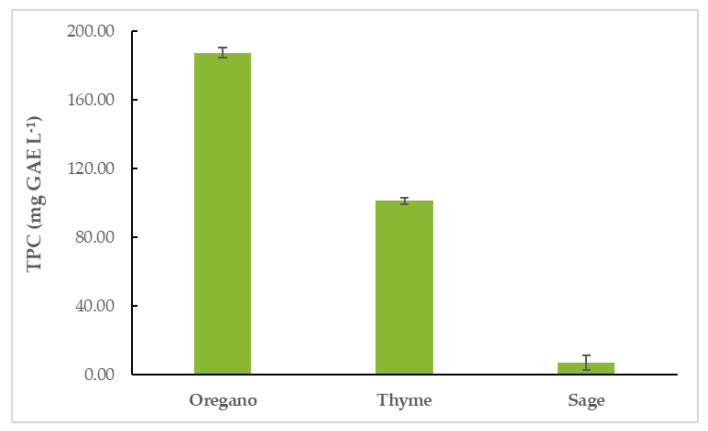
Total phenolic content (TPC) of EOs.

Oregano essential oil (OEO) had the highest TPC (187.64 ± 2.73 mg GAE L^−1^), followed by thyme (TEO) (101.36 ± 1.70 mg GAE L^−1^) and sage EO (SEO) (7.00 ± 4.19 mg GAE L^−1^). The results are shown in Figure 2 and Table S1.

### 3.4. Antioxidant Activity of EOs

#### 3.4.1. Interaction with DPPH and ABTS

**Figure 3:**
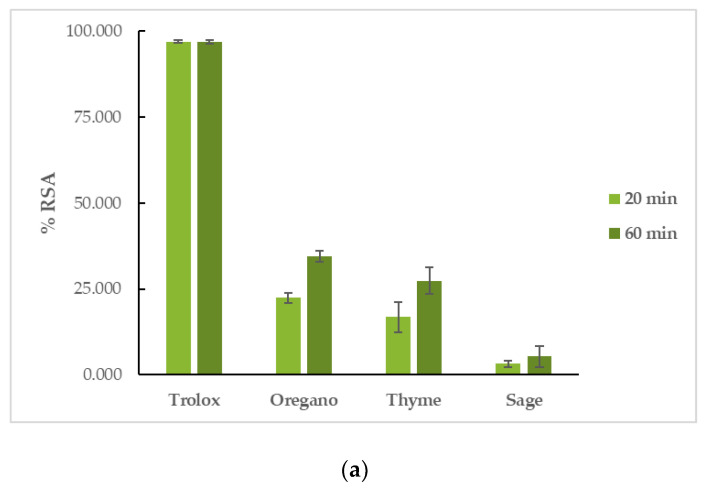

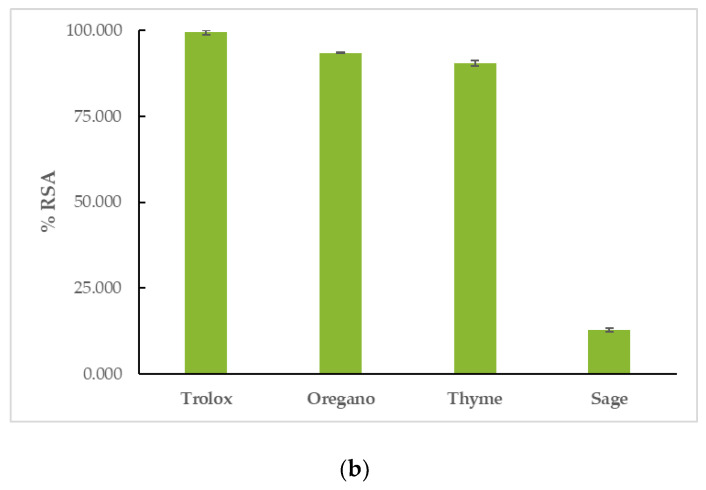
Antioxidant activity of EOs: interaction with (**a**) DPPH and (**b**) ABTS free radicals.

OEO had the highest reducing activity in both assays, followed by TEO, and finally, by SEO. More specifically, in the DPPH assay, OEO had a %RSA_20min_ = 22.46 ± 1.51% and %RSA_60min_ = 34.57 ± 1.55%, TEO had a %RSA_20min_ = 16.83 ± 4.46% and %RSA_60min_ = 27.38 ± 3.90%, and SEO had a %RSA_20min_ = 3.20 ± 0.92% and %RSA_60min_ = 5.31 ± 3.05%. The reaction proved to be time-dependent for the tested EOs, as their capacity to interact with the DPPH free radical increased with time. Trolox had a %RSA_20min_ = 97.00 ± 0.46% and %RSA_60min_ = 96.80 ± 0.53%. In the ABTS assay, OEO’s %RSA was 77.16 ± 0.51%, TEO’s %RSA was 73.38 ± 2.19%, and SEO’s %RSA was 7.24 ± 0.53%. Trolox had a %RSA = 99.41 ± 0.59%. The results can be seen in Figure 3 and Table S2.

#### 3.4.2. FRAP Assay

**Figure 4:**
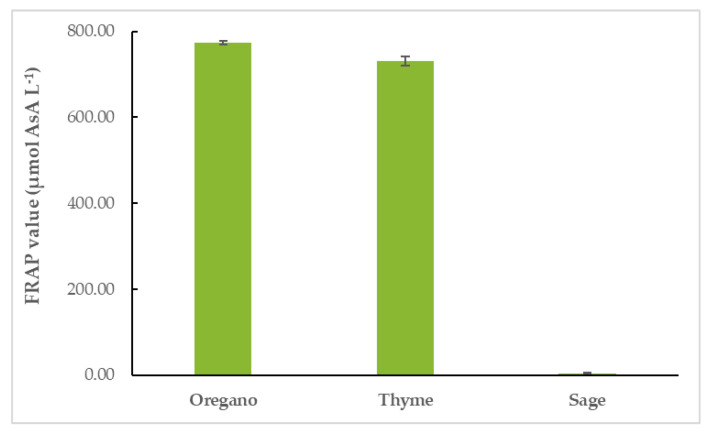
Antioxidant activity of EOs: FRAP values of EOs.

OEO had the highest FRAP value (774.04 ± 4.60 μmol AsA L^−1^), followed closely by TEO (731.29 ± 10.69 μmol AsA L^−1^). SEO’s FRAP value was the lowest of the three (4.30 ± 2.40 μmol AsA L^−1^), as shown in Figure 4 and Table S3.

### 3.5. Anti-Inflammatory (LOX-Inhibitory) Activity of EOs

**Figure 5:**
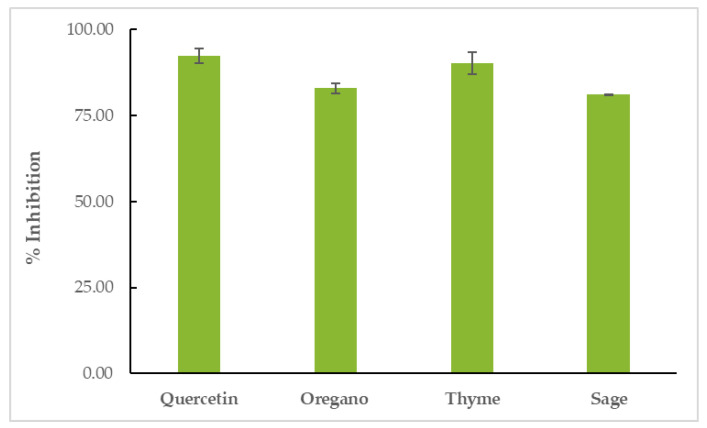
Inhibition of soybean LOX by EOs.

All EOs showed inhibitory activity against soybean lipoxygenase in vitro. TEO proved to be the most effective inhibitor of the enzyme (% LOX inhibition = 90.24 ± 3.24%), surpassed only by the positive control, quercetin (% LOX inhibition = 92.39 ± 2.21%). OEO’s inhibitory activity was 82.92 ± 1.40% and SEO’s inhibitory activity was 81.07 ± 0.24, as shown in Figure 5 and Table S4.

### 3.6. Antibacterial Capacity

The antibacterial activity was estimated using disk diffusion and minimum inhibitory concentration (MIC) assays. Briefly, the disk diffusion test was used to determine the susceptibility of ATCC bacterial strains to each of the investigated EOs. The effectiveness of each EO was based on the production of an inhibition zone, while an ineffective EO may not affect bacterial growth at all. MICs were defined as the lowest concentration of each EO that inhibited the visible growth of a microorganism after overnight incubation.

#### 3.6.1. Disk Diffusion Assay

**Figure 6:**
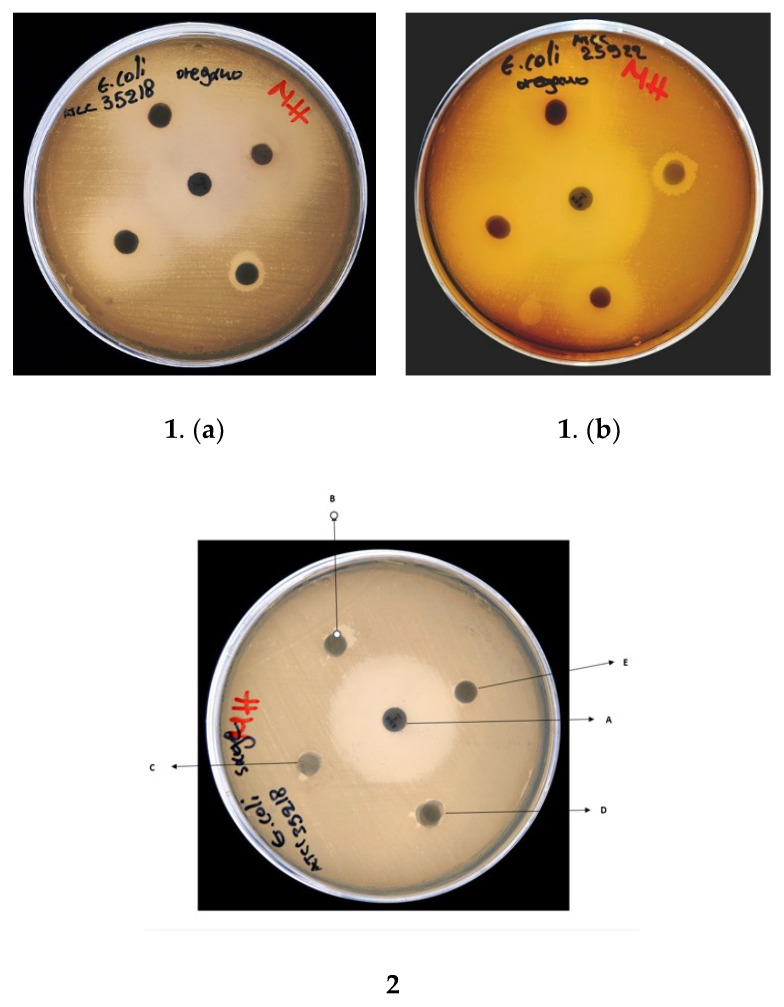
Disk diffusion assay showing antibacterial activity of: (**1**) oregano EO against (**a**) *E. coli* ATCC 35,218 and (**b**) *E. coli* ATCC 25922; (**2**) sage EO against *E. coli* ATCC 35218. An enrofloxacin disc (**A**) (5 mg/mL) used as positive control; EOS were used at concentrations of (**B**) 100%, (**C**) 50%, (**D**) 20%, and (**E**) 5%.

The results of the disk diffusion assays are shown in Table 4 and Figure 6. TEO exhibited the highest inhibitory activity among the three tested EOs, in some examples comparable to the control antibiotics. TEO contained a high carvacrol content. Sage, representing a low carvacrol concentration of 0.69%, showed the lowest inhibitory activity. In particular: (a) for the *S. aureus* ATCC 29,213 strain, TEO exhibited strong inhibition equal to OEO and control-1, (b) for the *E. coli* ATCC 25,922 strain, TEO had slightly stronger inhibition than ORE and less than Control-2, (c) for *E. coli* ATCC 35,218, TEO caused the same level of inhibition as Control-2 and slightly stronger than ORE, and (d) for *Lactobacillus fermentum* ATCC9398, TEO had the same level of inhibition compared to ORE and Control-1. In all cases, the inhibition of bacterial growth of EOs acted in a dose-dependent manner.

#### 3.6.2. Broth Microdilution Method

**Figure 7:**
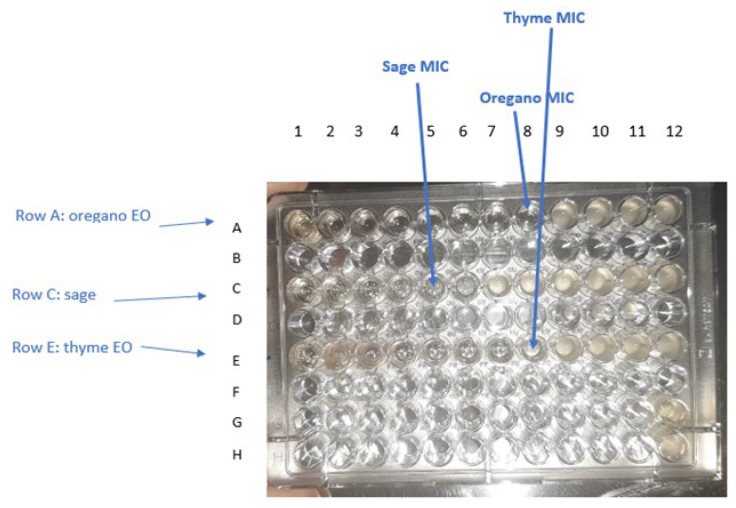
Determination of MIC for oregano, sage, and thyme EOs against *E. coli* ATCC 25,922 after incubation period by observing turbidity. The range of each EO concentration in the wells is 50% to 0.024% (*v*/*v*). Well 8 of row A shows no turbidity, therefore the concentration of oregano ΕO in that column was taken as the MIC value (0.39% *v*/*v*). Well 5 of row C shows no turbidity, therefore the concentration of sage ΕO in that column was taken as the MIC value (3.125% *v*/*v*). Well 8 of raw E shows no turbidity, therefore the concentration of thyme ΕO in that column was taken as the MIC value (0.39% *v*/*v*). Well G12 and H12 are the negative and positive control, respectively.

The EO MIC and MBC values (% *v*/*v*) against *S. aureus* ATCC 29213, *E. coli* ATCC 25922, *E. coli* ATCC 35218, and *L. fermentum* ATCC 9338 are presented in Table 5, along with the values of the aforementioned concentrations expressed as mg mL^−1^ (shown in Table 5). The results are displayed in Figure 7. While MIC is the lowest concentration of an antibacterial agent necessary to inhibit visible growth, MBC is the minimum concentration of an antibacterial agent that results in bacterial death. Thus, the closer the MIC to the MBC, the more bactericidal the compound [53].

For both values expressed in % *v*/*v* and mg mL^−1^, TEO had the best MIC and MBC values against *S. aureus* ATCC 29,213 compared to ORE, while SEO had no major effect. ORE presented the best results for *E. coli* ATCC 25922, *E. coli* ATCC 35,218, and *L. fermentum* ATCC 9338 strain. SEO had greater antimicrobial effects in *E. coli* ATCC 25,922 and *E. coli* ATCC 35,218 than *S. aureus* ATCC 29,213 or *L. fermentum* ATCC 9338 strains; however, its antimicrobial activity proved to be less than the other two EOs.

### 3.7. Antiparasitic Capacity

#### Anticoccidial Effect of EOs in *Eimeria Tenella* Using an In Vitro System

**Figure 8:**
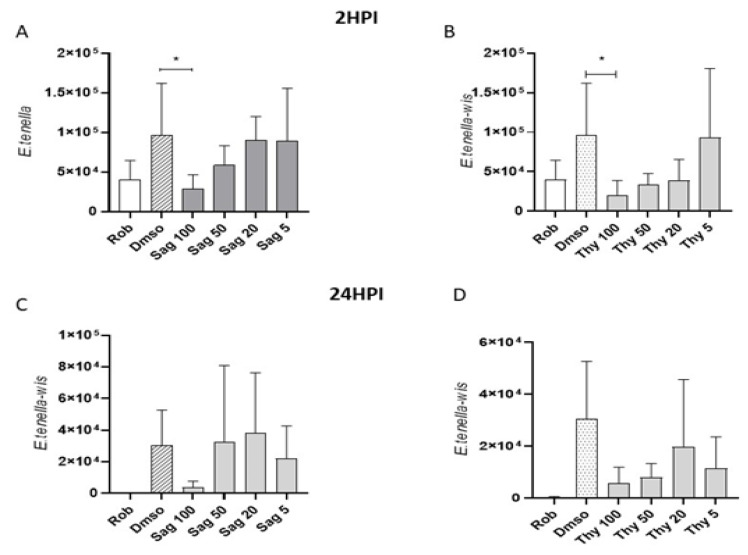
Effects of sage and thyme essential oils in *E. tenella* host cell invasion. Graphs show the number of intracellular sporozoites for: 2 (**A**,**B**) and 24 (**C**,**D**) hpi after pretreatment with sage (Sag) and thyme (Thy) essential oil at different concentrations (100, 50, 20, and 5 μg mL^−1^) and for the untreated (DMSO; 10 μL mL^−1^) and inhibition (robenidine, Rob; 5 μg mL^−1^) controls. *Asterisks indicate significant differences (Dunnett’s multiple comparisons test, *p* < 0.05).

Effects of thyme and sage essential oils in reducing host cell invasion by *E. tenella* sporozoites were evaluated in an in vitro system using quantitative qPCR and were compared to previous effects reported for OEO [30]. The effects were tested at two different time points after invasion (2 and 24 hpi) at different concentrations (100, 50, 20, and 5 μg mL^−1^). The untreated control (DMSO) and inhibition control (robenidine) of invasion were included. Normal morphology was observed in MDBK cells exposed to concentrations of 100 μL mL^−1^ of oregano, thyme, or sage EOs. The invasion of *E. tenella* in the controls showed the expected effect (increase and decrease of infection for DMSO and robenidine, respectively). At 2 hpi, only the highest concentration of sage and thyme (100 μg mL^−1^) showed a significant reduction in comparison to the untreated control (DMSO vs. sage 100, thyme 100, Dunnett’s multiple comparisons test, *p* < 0.05) (Figure 8A,B). At 24 hpi, none of the tested concentrations for sage and thyme showed a significant reduction in relation to the untreated control (DMSO). However, there was a tendency towards the reduction of invaded sporozoites (Table 6, Figure 8C,D).

## 4. Discussion

In the current study, oregano, thyme, and sage EOs were examined for their in vitro antioxidant, anti-inflammatory, antimicrobial, and antiparasitic efficacy. Based on the GC-MS results, the main bioactive compounds of the oregano EO were carvacrol and γ-terpinene, of thyme EO were thymol and p-cymene, and of sage EO were eucalyptol and camphor. Essential oils have already been used in veterinary medicine and may be classified as follows: oils attracting and repelling animals; insecticidal, pest-repellent, and antiparasitic oils; oils used in animal feed; and oils used in the disease treatment of animals [54].

Although their mechanisms of action have not been fully elucidated, Many EOs have been widely applied to treat infections due to their antimicrobial activity [55]. The results from the in vitro studies in this work showed that the essential oils inhibited bacterial growth and parasite invasion with varied effectiveness. The antioxidant effects were evaluated using three different tests providing comparisons for different antioxidant mechanisms. The significance of EOs in combating oxidative stress in cells and organisms, together with their antimicrobial, anti-inflammatory, and other functions, has been validated by many studies; however, only a few have conducted direct comparisons [30,56]. There are many methods measuring the antioxidant activity of OEO, TEO, and SEO components, usually based on the scavenging of free radical species, such as DPPH, ABTS, and FRAP, determining the absorbance of the reduced product using a photometric assay. Here, the three tested EOs were evaluated and proved to be potent LOX inhibitors as well. In this study, oregano EO showed the highest antioxidant activity, both as a reducing agent and a radical scavenger. In an earlier extensive study, it was reported that Mediterranean aromatic plants and spices, such as annatto, cumin, oregano, sweet and hot paprika, rosemary, and saffron, which are traditionally used for their aromatic properties in the preparation of Mediterranean food, exhibited strong antioxidant activity as scavengers of several reactive oxygen species. This study provides evidence in support of replacing synthetic antioxidants with natural spice extracts that could further enrich characteristic colors and flavors, encouraging the use of these spices in the design of new functional foods [56].

The anti-inflammatory effect was also tested by the lipoxygenase (LOX) inhibition assay. The practical value of in vitro screening and the value added by techniques such as the LOX inhibition assay was first recommended almost two decades ago [57], aiming to develop safer anti-inflammatory drugs and to supplement in vivo testing. The authors reviewed a large number of active plant extracts and compounds identified from a limited sampling of Africa’s medicinal flora, further emphasizing potential areas for the identification of the biological activity of plant extracts. It was highlighted that impurities, poor technique, or partial isolation of active components can hinder drug development. Routine testing for the toxicity, selectivity, and stability of compounds were presented as critical attributes of compounds considered for drug development, both for alternative human medicine and animal health care.

In the present study, thyme EO was observed to be the strongest inhibitor of soybean lipoxygenase, in accordance with the existing literature. Wei and Shibamoto [58] reported that the essential oil of *Thymus vulgaris* exerted a particularly powerful antioxidant and LOX-inhibitory action. Abdelli et al. [59], who studied the potential of Algerian TEO to mitigate carrageenan-induced paw edema in mice, also reported the inhibition of the inflammatory process, an action attributed mainly to thymol. On the other hand, carvacrol, another major constituent of TEO, was reported by Hotta et al. [60] to be an inhibitor of COX-2 via the activation of PPARα and γ. It is known that COX-2 is a key enzyme involved in prostaglandin biosynthesis and therefore in the inflammatory response, whose expression seems to be controlled by PPARα and γ in smooth muscle cells and macrophages, respectively. There is extended literature to support the suggestion that all three EOs have the potential to be employed as anti-inflammatory agents and alternatives to synthetic antioxidants in food preparations. For example, Leyva-López et al. [61] demonstrated that terpenes, such as thymol and carvacrol acetate, obtained from three Mexican oregano species, *Lippia graveolens, Lippia palmeri*, and *Hedeoma patens*, significantly reduced the levels of ROS and NO produced by macrophage cells stimulated with lipopolysaccharide (LPS). Furthermore, EOs of *Origanum majorana* (10 µg mL^−1^) reduced the production of tumor necrosis factor-alpha (TNF-α), interleukin-1β (IL-1β), and IL-6 in LPS-activated THP-1 human macrophage cells [62]. Recently, Han and Parker [63] showed that EOs obtained from *O. vulgare* significantly inhibited levels of the inflammatory biomarkers monocyte chemoattractant protein-1 (MCP-1), vascular cell adhesion molecule-1 (VCAM-1), and intracellular cell-adhesion molecule-1 (ICAM-1) in activated primary human neonatal fibroblasts. These findings suggest that OEOs have anti-inflammatory properties. The individual components of EOs from oregano have also been studied to better understand their effect on inflammation. Thus, Lima et al. [64] demonstrated that carvacrol exerts anti-inflammatory activity in a mouse inflammation model. When carvacrol was administrated to mice (at 50 and 100 mg kg^−1^) presenting paw edema, the levels of IL-1β and prostaglandin E2 (PGE2) prostaglandins were diminished. The anti-inflammatory effect of carvacrol is due both to the reduction of pro-inflammatory mediators and the increase of anti-inflammatory cytokines (IL-10) [64]. Other EO components, such as p-cymene and *β*-caryophyllene [63,65,66,67], have also demonstrated anti-inflammatory properties. The studies mentioned above indicate that diverse oregano species might be used as anti-inflammatory agents and could be added to formulations for the prevention or treatment of inflammation-related diseases. Nevertheless, and since OEOs might exert toxic effect on cells, several in vivo and clinical studies are needed before the EOs can be used as an alternative to treat inflammation [68]. *Salvia triloba* antioxidant activity is due to high levels of phenolics, terpenoids, polyphenols, and flavonoids, as revealed by in vitro and in vivo studies [38,40,69].

Studies on the antimicrobial properties of essential oils against microorganisms with veterinary importance in vitro and in vivo are still limited. Rusenova and Parvanov [54] evaluated twelve essential oils for their inhibitory activity against some microorganisms of veterinary interest using disk diffusion and the most active were selected for further study using the agar dilution method. Disk diffusion showed variation in the antimicrobial activity of selected essential oils. According to the agar dilution method, the most potent essential oils were cinnamon, oregano, lemongrass, and thyme. MICs were tested at concentrations ranging from 2.0 to 0.008% (*v*/*v*). These inhibitory effects are interesting in relation to treatment of bacterial and yeast infections in animals [55]. Moreover, the inhibitory effects of sage and oregano essential oils against *E. coli* were noted when these EOs were applied in meat preparations such as minced beef [69].

Finally, in this study, the in vitro anticoccidial activity of oregano, thyme, and sage essential oils was evaluated, based on their effect on the inhibition of coccidial (*E. tenella*) invasion in MDBK cells along with their cytotoxic effects. Previous results showed that oregano possesses very strong anticoccidial activity in vitro, evidenced by the inhibition of sporozoite invasion at the higher concentrations tested, potentially caused by a toxic effect that left few parasites fit to invade cells [30]. These results were similar to those previously reported for sporozoites pretreated with oregano essential oil [30], where only the highest concentration (100 μg mL^−1^) showed a significant reduction in the number of sporozoites to the untreated control at 2 hpi, despite a clear reduction tendency between groups. Following identical protocols, sage and thyme present a potent inhibitory effect on sporozoite invasion. Both had a noticeable reduction at higher concentrations, with thyme showing more consistent effects at lower concentrations. Oregano essential oil exhibited an effect comparable with robenidine, a well-known anticoccidial drug. The same high essential oil concentration did not show any deleterious effects on the host cells based upon a microscopic assessment of the cell morphology within the monolayer. Cytotoxic effects on the host cells could have affected parasite invasion and proliferation [70]. Sporozoites have been shown to begin endogenous development into schizonts from 28 hpi [71]. Further studies to evaluate the effects of the tested essential oils in this part of the eimerian lifecycle, and the extent to which the pretreatment of free sporozoites has an effect would be of great interest. Although the mode(s) of action or mechanisms involved have not been elucidated, a reasonable explanation for this anticoccidial activity is the hydrophobic character and low molecular weight of the main phenolic compounds present in these essential oils that might allow them to disintegrate the outer cell membranes [72]. This may cause an increase in cytoplasmic membrane permeability and lead to cell death caused by the leakage of ions, energy loss, and the diffusion of cell contents [14]. Furthermore, the high lipid solubility of oregano and other essential oils is likely to permit rapid diffusion through parasite and host cell membranes. Other possible mechanisms include interference with the calcium-mediated signaling that is a necessary mechanism for invasion by *E. tenella* sporozoites [72]. The hydrophobic character of these compounds may suggest interaction with membrane components and permeability [73].

It is believed that the hydrophobic compounds in EOs may penetrate bacterial and parasitic cells causing cell deformities and organelle dysfunction [74]. If the carvacrol concentration increases, then more molecules interact with the phospholipid bilayer, upsetting the membrane fluidity [75,76]. Accordingly, carvacrol, thymol, and the major bioactive ingredients of the EOs tested here may also exert a toxic effect on the upper layer of mature enterocytes of the intestinal mucosa. Recent scientific research has shown that many plants used as food or in traditional medicine are potentially toxic, mutagenic, and carcinogenic [77,78,79,80,81]. For this reason, in this study, their in vitro cytotoxic effects on MDBK cells were assessed and were detected to be very low. Moreover, other studies have demonstrated that MDBK cells are able to produce cytokines when stimulated by exposure to viruses, suggesting that an immune response could also be involved in the anticoccidial effect of these metabolite compounds of the plants in question [28,30,82]. Traditionally used medicinal plants are assumed to be safe based on their long usage in the treatment of various ailments, according to knowledge accumulated over centuries.

The results of this study are in agreement with several studies conducted on natural plant essential oils to indicate their in vitro antioxidant and antimicrobial properties, justifying their potential use in industrial applications, as, for example, *Melaleuca alternifiolia* [83]. Renewed interest in traditional pharmacopeias means that researchers are concerned not only with determining the scientific rationale for the plant’s usage, but also the discovery of novel compounds of pharmaceutical value. Instead of relying on trial and error, as in random screening procedures, traditional knowledge helped scientists to target plants that may be medicinally useful. An estimated 122 drugs from 94 plant species have already been discovered through ethnobotanical leads. Through the last two decades, 33% of the 1394 small molecules that were approved as new drugs were either natural products or natural derivatives, and another 35% were created around a natural pharmacophore acknowledging their significance. Various assays can be used to test for biological activity, firstly in vitro and later, for promising natural products, in vivo. Crude or fractionated extracts and sometimes individual compounds have been screened for antibacterial, anti-inflammatory, antioxidant, anthelmintic, anti-amoebic, antischistosomal, and/or antimalarial activity, as well as psychotropic and neurotropic properties.

## 5. Conclusions

This study confirms the potent antioxidant, anti-inflammatory, and antimicrobial (both antibacterial and anticoccidial) activity of selected essential oils derived from oregano, thyme, and sage. These oils also exhibited very low cytotoxic activity. Oregano and thyme oils, according to the agar dilution method, were the most effective. Thyme presented the highest anti-inflammatory effect, and the antioxidant activity was in the order of oregano > thyme > sage. Together, these essential oils can comprise an effective combination that could be further tested in vivo by challenging broilers with coccidia, *E. coli*, and other pathogens and may potentiate the efficacy of chemotherapeutics/prophylactics with economic relevance to broiler rearing. Bioactive compounds derived from natural resources such as oregano, thyme, and sage plants merit great interest due to their pharmacological and medicinal properties, low adverse effects, and economic value. Although it is difficult to extrapolate the doses employed in vitro to in vivo, further work needs to be undertaken to determine the appropriate doses of essential oils showing both antimicrobial activity and very low detrimental effect on animal cells.

## Figures and Tables

**Table 1:** Chemical composition of oregano essential oil (OEO).

Compounds ^1^	Retention Time (min)	%	Identification Method ^2^
α-Pinene	4.399	0.52	RT, MS, Co-GC
α-Thujene	4.534	0.83	RT, MS, Co-GC
Camphene	5.510	0.07	RT, MS
α-Phellandrene	9.863	0.23	RT, MS
β-Myrcene	10.210	2.03	RT, MS, Co-GC
α-Terpinene	10.808	1.84	RT, MS, Co-GC
γ-Terpinene	16.641	10.59	RT, MS, Co-GC
p-Cymene	19.608	8.90	RT, MS, Co-GC
1-Octen-3-ol	34.963	0.61	RT, MS
*cis*-Sabinenehydrat	35.365	0.34	RT, MS
β-Caryophyllene	40.800	1.01	RT, MS, Co-GC
1-Terpinen-4-ol	41.688	0.37	RT, MS, Co-GC
Thymol methyl ether	41.829	0.21	RT, MS
Borneol	45.772	0.40	RT, MS, Co-GC
β-Bisabolene	46.715	0.41	RT, MS, Co-GC
Thymol	62.680	3.69	RT, MS, Co-GC
Carvacrol	63.512	67.95	RT, MS, Co-GC
Total essential oil yield (mL OEO 100 g^−1^ dry herb) = 5.49 ± 0.07			

^1^ Compounds listed in order of elution from an INNOWAX capillary column. ^2^ Identification method: RT = retention time, MS = mass spectrum, Co-GC = co-injection with authentic compound.

**Table 2:** Chemical composition of thyme essential oil (TEO).

Compounds ^1^	Retention Time (min)	%	Identification Method ^2^
α-Pinene	4.417	0.99	RT, MS, Co-GC
Camphene	5.354	0.21	RT, MS, Co-GC
α-Phellandrene	9.517	0.11	RT, MS
Myrcene	9.865	0.98	RT, MS
α-Terpinene	10.438	0.66	RT, MS, Co-GC
Limonene	11.737	0.08	RT, MS, Co-GC
β-Phellandrene	12.349	0.07	RT, MS
γ-Terpinene	15.989	2.03	RT, MS, Co-GC
p-Cymene	18.854	5.00	RT, MS, Co-GC
1-Octen-3-ol	34.646	0.40	RT, MS
Linalool	39.496	1.42	RT, MS, Co-GC
β-Caryophyllene	40.440	2.27	RT, MS, Co-GC
Terpinen-4-ol	41.392	0.72	RT, MS, Co-GC
Borneol	45.460	1.81	RT, MS, Co-GC
Caryophyllene oxide	55.279	0.23	RT, MS, Co-GC
Thymol	62.350	0.98	RT, MS, Co-GC
Guaiol	62.769	0.49	RT, MS
Carvacrol	63.156	80.68	RT, MS
Apiol	67.128	0.62	RT, MS
Total essential oil yield (mL TEO 100 g^−1^ dry herb) = 4.15 ± 0.15			

^1^ Compounds listed in order of elution from an INNOWAX capillary column. ^2^ Identification method: RT = retention time, MS = mass spectrum, Co-GC = co-injection with authentic compound.

**Table 3:** Chemical composition of sage essential oil (SEO).

Compounds ^1^	Retention Time (min)	%	Identification Method ^2^
α-Pinene	4.330	5.90	RT, MS, Co-GC
Camphene	5.405	3.97	RT, MS, Co-GC
β-Pinene	6.760	1.62	RT, MS, Co-GC
β-Myrcene	9.989	3.50	RT, MS
α-Terpinene	10.555	0.11	RT, MS, Co-GC
D-Limonene	11.901	1.71	RT, MS, Co-GC
Eucalyptol	12.721	55.84	RT, MS, Co-GC
γ-Terpinene	16.172	0.05	RT, MS, Co-GC
3-Octanone	17.964	0.09	RT, MS
p-Cymene	19.084	1.53	RT, MS, Co-GC
Octen-1-ol, acetate	30.336	0.07	RT, MS
*cis*-Thujone	31.641	1.87	RT, MS, Co-GC
*trans*-thujone	32.852	0.76	RT, MS, Co-GC
Isopropenyl toluene	33.090	0.05	RT, MS
cis-Linaloloxide	33.691	0.02	RT, MS
1-Octen-3-ol	34.753	0.31	RT, MS
Camphor	36.630	9.82	RT, MS, Co-GC
Linalool	39.602	0.59	RT, MS, Co-GC
Linalool acetate	39.743	0.16	RT, MS, Co-GC
Bornyl acetate	40.241	0.45	RT, MS, Co-GC
β-Caryophyllene	40.565	0.37	RT, MS, Co-GC
Fenchol	40.765	0.04	RT, MS
Aromadendrene	40.993	0.11	RT, MS
Terpinen-4-ol	41.502	0.54	RT, MS
α-Caryophyllene	43.727	0.08	RT, MS, Co-GC
Myrcenol	44.572	1.07	RT, MS
α-Terpineol acetate	45.331	0.95	RT, MS
α-Terpineol	45.604	4.61	RT, MS, Co-GC
Myrtenol	49.163	0.10	RT, MS
Nerol	51.464	0.03	RT, MS
Caryophyllene oxide	55.424	0.12	RT, MS, Co-GC
Ledol	58.990	0.65	RT, MS
Carvacrol	63.317	0.69	RT, MS
Aromadendrene oxide	65.522	0.08	RT, MS
Epi-13-Manool	75.594	0.15	RT, MS
Total essential oil yield (mL SEO 100 g^−1^ dry herb) = 4.66 ± 0.24			

^1^ Compounds listed in order of elution from an INNOWAX capillary column. ^2^ Identification method: RT = retention time, MS = mass spectrum, Co-GC = co-injection with authentic compound.

**Table 4:** Antibacterial activity of EOs against bacterial ATCC strains by paper disk diffusion method.

Bacterial ATCC Strain	EO Concentration	Essential Oils		
		OEO	TEO	SEO
		Diameter of Inhibition Zone (mm)		
*S. aureus* ATCC 29213	* C = 100%	30	30	18
	C = 50%	30	30	18
	C = 20%	22	26	18
	C = 5%	12	12	0
	Control_1	30	30	30
*E. coli* ATCC 25922	C = 100%	25	28	10
	C = 50%	25	22	8
	C = 20%	20	18	0
	C = 5%	12	12	0
	Control_2	33	33	33
*E. coli* ATCC 35218	C = 100%	28	30	0
	C = 50%	25	26	0
	C = 20%	25	20	0
	C = 5%	8	14	0
	Control_2	30	30	30
*Lactobacillus fermentum* ATCC 9338	C = 100%	35	35	8
	C = 50%	35	35	8
	C = 20%	35	35	6
	C = 5%	35	35	0
	Control_1	35	35	35

1 = Control 1, penicillin G (10 μg, Oxoid, Basingstoke, UK); 2 = Control 2, enrofloxacin (5 μg, Oxoid, Basingstoke, UK); * C = 100% means that pure EO (100% concentration, no diluted with DMSO) was impregnated the sterile filter disk; C = 50% means that 50 volumes of each EO were diluted in 50 volumes of DMSO; C = 20% means that 20 volumes of each EO were diluted in 80 volumes of DMSO; and C = 5% means that 5 volumes of each EO were diluted in 95 volumes of DMSO.

**Table 5:** MIC and MBC values (mg mL^−1^) of EOs against *S. aureus* ATCC 29213, *E. coli* ATCC 25922, *E. coli* ATCC 35218, and *L. fermentum* ATCC 9338.

Bacterial Strains	Essential Oils					
	OEO		TEO		SEO	
	MIC (mg mL^−1^)	MBC (mg mL^−1^)	MIC (mg mL^−1^)	MBC (mg mL^−1^)	MIC (mg mL^−1^)	MBC (mg mL^−1^)
*S. aureus* ATCC 29213	0.123	0.246	0.018	0.036	2.250	4.500
*E. coli* ATCC 25922	0.031	0.031	0.036	0.071	2.812	0.563
*E. coli* ATCC 35218	0.031	0.031	0.071	0.071	0.563	1.125
*L. fermentum* ATCC 9338	0.031	0.031	0.071	0.071	2.250	2.250

MIC: Minimum inhibitory concentration; MBC: minimum bactericidal concentration.

**Table 6:** Sporozoite inhibition after treatment with oregano, sage, and thyme EOs at various concentrations.

Time Point	Pretreatment	100 μg mL^−1^	50 μg mL^−1^	20 μg mL^−1^	5 μg mL^−1^
2 hpi	OEO	82.8 ± 6.95	15.5 ± 37.1	0 ± 0	0 ± 0
	TEO	81.3 ± 14.1	62.8 ± 24.6	62.9 ± 20.3	24.1 ± 57.1
	SEO	72.2 ± 18.4 *	33.0 ± 44.8	11.6 ± 35.0	5.25 ± 14.6
24 hpi	OEO	92.9 ± 6.9	81.5 ± 25.6	38.1 ± 13.1	33.3 ± 66.7
	TEO	90.8 ± 17.9	73.1 ± 1.6	49.6 ± 54.9	67.4 ± 18.5
	SEO	89.6 ± 9.4	32.5 ± 65.1	31.6 ± 63.3	38.5 ± 42.5

* Average of the biological replicates ± range.

## Data Availability

Not applicable.

## Supplementary Materials

The following supporting information can be downloaded at: https://www.mdpi.com/article/10.3390/life12111783/s1, Figure S1. GC-MS chromatogram of oregano essential oil (OEO); Figure S2. GC-MS chromatogram of thyme essential oil (TEO); Figure S3. GC-MS chromatogram of sage essential oil (SEO); Table S1. Total phenolic content (TPC) of EOs; Table S2. Antioxidant activity of EOs: Interaction with DPPH and ABTS radicals; Table S3. Antioxidant activity of EOs: FRAP values; Table S4. Inhibition of soybean LOX by EOs.
