# Supplementary material for: In Vitro Antioxidant, Antimicrobial, Anticoccidial, and Anti-Inflammatory Study of Essential Oils of Oregano, Thyme, and Sage from Epirus, Greece
Source: Life (Basel). 2022 Nov 4;12(11):1783. doi: 10.3390/life12111783 (PMC9693314; doi:10.3390/life12111783)
Supplement: Supplementary file 1 [file life-12-01783-s001.zip › life-1974680-supplementary.pdf]

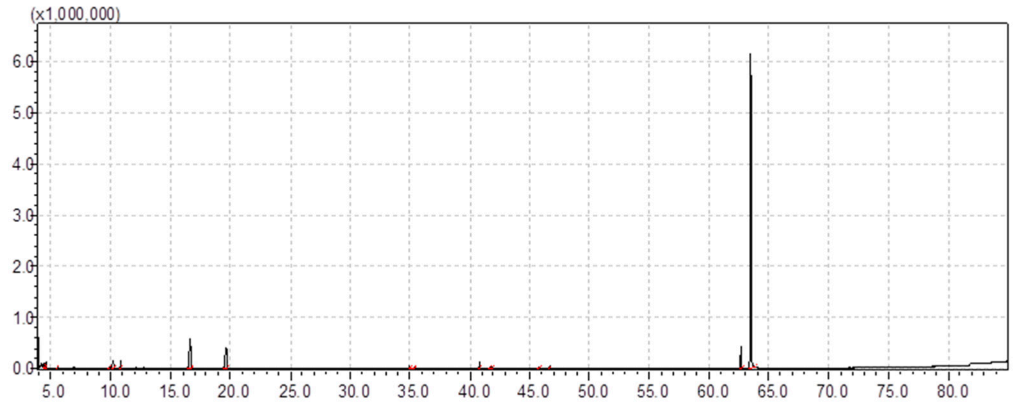

Figure S1. GC-MS chromatogram of oregano essential oil (OEO)

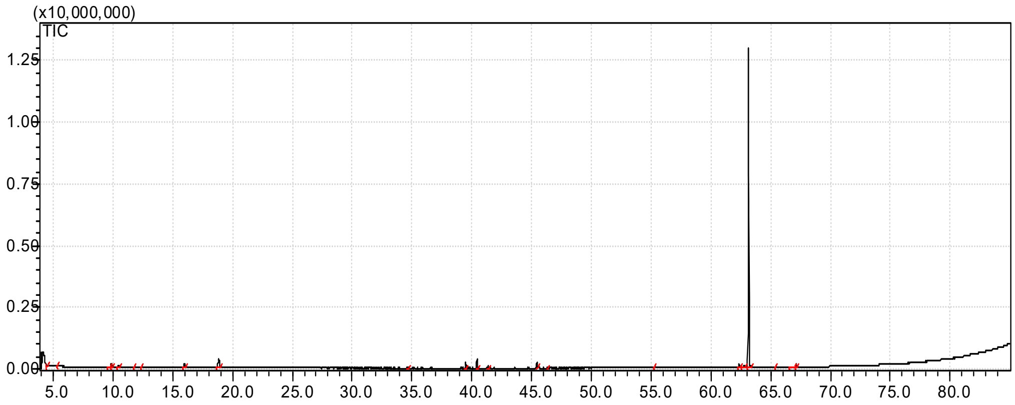

Figure S2. GC-MS chromatogram of thyme essential oil (TEO)

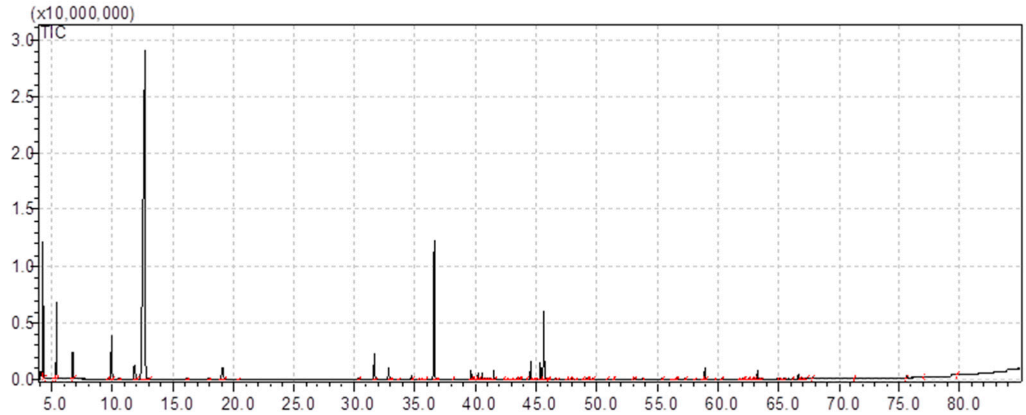

Figure S3. GC-MS chromatogram of sage essential oil (SEO)

Table S1. Total phenolic content (TPC) of EOs

**Sample (5 mg mL^{-1})**	**TPC (mg GAE L^{-1})**
Oregano	187.64 ± 2.73
Thyme	101.36 ± 1.70
Sage	7.00 ± 4.19

Table S2. Antioxidant activity of EOs: Interaction with DPPH and ABTS radicals

**Sample**	**DPPH (5 mg mL^{-1})**		**ABTS (1 mg mL^{-1})**
	% RSA (20 min)	% RSA (60 min)	% RSA
Trolox (5 mM)	97.01 ± 0.46	96.80 ± 0.53	99.41 ± 0.59
Oregano	22.46 ± 1.51	34.57 ± 1.55	77.16 ± 0.51
Thyme	16.83 ± 4.46	27.38 ± 3.90	73.38 ± 2.19
Sage	3.20 ± 0.92	5.31 ± 3.05	7.24 ± 0.53

Table S3. Antioxidant activity of EOs: FRAP values

**Sample (1 mg mL^{-1})**	**FRAP value (μmol AsA L^{-1})**
Oregano	4.30 ± 2.40
Thyme	731.29 ± 10.69
Sage	774.04 ± 4.60

Table S4. Inhibition of soybean LOX by EOs

**Sample (0.5 mg mL^{-1})**	**% LOX inhibition**
Quercetin (1 mg mL^{-1})	92.39 ± 2.21
Oregano	82.92 ± 1.40
Thyme	90.24 ± 3.24
Sage	81.07 ± 0.24
